# Mycoprotein ingestion within or without its wholefood matrix results in equivalent stimulation of myofibrillar protein synthesis rates in resting and exercised muscle of young men

**DOI:** 10.1017/S0007114522003087

**Published:** 2023-07-14

**Authors:** Sam West, Alistair J. Monteyne, Gráinne Whelehan, Doaa R. Abdelrahman, Andrew J. Murton, Tim J. A. Finnigan, Jamie R. Blackwell, Francis B. Stephens, Benjamin T. Wall

**Affiliations:** 1 Department of Sport and Health Sciences, College of Life and Environmental Sciences, Heavitree Road, University of Exeter, Exeter, UK; 2 Department of Surgery, University of Texas Medical Branch, Galveston, TX, USA; 3 Sealy Center of Aging, University of Texas Medical Branch, Galveston, TX, USA; 4 Marlow Foods Ltd, Station Road, Stokesley, NYK, UK

**Keywords:** Mycoprotein, Wholefood, Muscle protein synthesis, Resistance exercise

## Abstract

Ingestion of mycoprotein stimulates skeletal muscle protein synthesis (MPS) rates to a greater extent than concentrated milk protein when matched for leucine content, potentially attributable to the wholefood nature of mycoprotein. We hypothesised that bolus ingestion of mycoprotein as part of its wholefood matrix would stimulate MPS rates to a greater extent compared with a leucine-matched bolus of protein concentrated from mycoprotein. Twenty-four healthy young (age, 21 ± 2 years; BMI, 24 ± 3 kg.m^2^) males received primed, continuous infusions of L-[*ring*-^2^H_5_]phenylalanine and completed a bout of unilateral resistance leg exercise before ingesting either 70 g mycoprotein (MYC; 31·4 g protein, 2·5 g leucine; *n* 12) or 38·2 g of a protein concentrate obtained from mycoprotein (PCM; 28·0 g protein, 2·5 g leucine; *n* 12). Blood and muscle samples (*vastus lateralis*) were taken pre- and (4 h) post-exercise/protein ingestion to assess postabsorptive and postprandial myofibrillar protein fractional synthetic rates (FSR) in resting and exercised muscle. Protein ingestion increased plasma essential amino acid and leucine concentrations (*P* < 0·0001), but more rapidly (both 60 *v*. 90 min; *P* < 0·0001) and to greater magnitudes (1367 *v*. 1346 μmol·l^–1^ and 298 *v*. 283 μmol·l^–1^, respectively; *P* < 0·0001) in PCM compared with MYC. Protein ingestion increased myofibrillar FSR (*P* < 0·0001) in both rested (MYC, Δ0·031 ± 0·007 %·h^–1^ and PCM, Δ0·020 ± 0·008 %·h^–1^) and exercised (MYC, Δ0·057 ± 0·011 %·h^–1^ and PCM, Δ0·058 ± 0·012 %·h^–1^) muscle, with no differences between conditions (*P* > 0·05). Mycoprotein ingestion results in equivalent postprandial stimulation of resting and post-exercise myofibrillar protein synthesis rates irrespective of whether it is consumed within or without its wholefood matrix.

Dietary protein is essential to support the maintenance of skeletal muscle mass and to facilitate muscle reconditioning and/or tissue accrual in response to exercise. This is accomplished by the ingestion of dietary protein stimulating muscle protein synthesis (MPS) rates, resulting in postprandial muscle protein accretion^(1)^. The postprandial increase in MPS rates is achieved by a transient rise in circulating amino acids following protein ingestion, which act as both stimulus and substrate^(1)^. Although essential amino acids are the bulk drivers of postprandial MPS rates^(2)^, the relative increase in MPS is often suggested to be primarily dictated by rapid and/or large postprandial rises in plasma leucine concentrations (coined ‘the leucine trigger’ or ‘threshold’ hypothesis^(3,4)^). This notion of the nutritional regulation of postprandial MPS has helped understanding of key parameters of protein nutrition, such as optimal amounts^(5,6)^, timing^(7,8)^ and daily distribution^(7)^, to maximise MPS responses to *isolated* proteins. However, recent data imply that when protein is consumed within a wholefood source, the role that leucine plays in the postprandial regulation of MPS is more nuanced^(9,10)^.

Mycoprotein is a fungal-derived protein-rich (approximately 45 %) wholefood source, produced by continuous flow fermentation of the filamentous fungi *Fusarium venenatum.* We reported that mycoprotein ingestion provides a bioavailable source of amino acids but, due to being a fibre-rich wholefood, a relatively slow (and low) plasma leucine response^(11)^. Nevertheless, we recently showed that bolus ingestion of mycoprotein increases MPS rates in rested and exercised muscle to a greater extent than a leucine-matched bolus of concentrated milk protein^(12)^. While the multitude of differences between two entirely different protein sources precluded our being able to determine the causative mechanism, the data ran contrary to the leucine trigger hypothesis and implied non-protein constituents may be involved.

In line with others’ interpretations^(9,10)^, we have speculated such data may be attributable to a dietary protein’s anabolic potency being augmented when present within its specific wholefood matrix. In support, ingestion of whole eggs^(13)^ or whole milk^(14)^ results in greater anabolic responses compared with their isolated counterparts (egg whites and skimmed milk, respectively), despite the wholefood forms eliciting slower and lower plasma leucine responses^(13)^. Contrary to these data, casein co-ingested with a serum milk matrix did not increase MPS rates compared with isolated casein^(15)^, perhaps highlighting a subtle difference from ingesting protein *within* its natural wholefood as opposed to *with added* constituent parts (i.e. ‘co-ingestion’). What is presently lacking from the literature are any comparisons of the MPS response to non-animal-derived protein sources within and without natural wholefood matrices.

In the present study, we hypothesised that the MPS response to bolus mycoprotein ingestion is more contingent on its existence within a wholefood matrix than its ability to elicit rapid or high levels of postprandial leucinaemia. To test this hypothesis, we compared the myofibrillar protein synthesis (MyoPS) response to bolus ingestion of a novel dietary protein concentrate extracted from mycoprotein (PCM; 73 % protein) to that of a naturally produced mycoprotein (MYC; 45 % protein) in resting and exercised muscle of healthy young men.

## Methods

### Participants

Twenty-four resistance-trained, young and healthy men volunteered to take part in the present study (age, 21 ± 1 years; body mass, 78 ± 2 kg; BMI, 24 ± 1 kg.m^2^; 22 Caucasian and 2 Mixed race). Participants’ characteristics are presented in Table 1. Participants were considered resistance-trained if they were engaged in resistance training > 3 times per week for > 3 months prior to taking part in the study. This population was selected as training status impacts the anabolic response to exercise^(16)^. Therefore, selecting resistance-trained individuals ensured optimal exercise execution, (more) ecological validity (to exercise training) and an assumed greater homogeneity of responses to exercise. Subjects had not undergone any previous stable isotope tracer protocols in the previous 6 months, ensuring negligible background stable isotope enrichments. Exclusion criteria included any metabolic impairment, cardiovascular complications or allergies to mycoprotein/Quorn/edible fungi or environmental moulds. Subjects were enrolled in the study after being deemed healthy based on blood pressure (< 140/90 mmHg), BMI (18–30 kg/m^2^) and responses to a routine medical health questionnaire. Experimental procedures, potential risks and the purpose of the study were explained to the participants prior to obtaining informed written consent. This study was approved by the Sport and Health Sciences ethics committee of the University of Exeter (190 206/B/07) in accordance with the Declaration of Helsinki and is registered at ClinicalTrials. Gov (NCT04084652). Recruitment and data collection were carried out in the Nutritional Physiology Research Unit at the University of Exeter between April 2019 and December 2019.

**Table 1.** Participants’ characteristics (Mean values and standard errors of the mean)

	MYC (*n* 12)		PCM (*n* 12)	
	Mean	sem	Mean	sem
Age (years)	21·3	1	21·5	1
Height (cm)	179·5	2	178·1	2
Weight (kg)	77·8	3	77·2	2
BMI (kg/m^2^)	24·1	1	24·3	1
Systolic blood pressure (mmHg)	127	1	126	2
Diastolic blood pressure (mmHg)	70	2	68	2
Fat (% body mass)	11	2	10	1·3
Lean mass (kg)	69	3	70	2
Leg press 1RM (dominant) (kg)	157	10	159	9
Leg press 1RM (non-dominant) (kg)	152	9	156	8
Leg extension 1RM (dominant) (kg)	61	3	66	4
Leg extension 1RM (non-dominant) (kg)	61	3	66	4

MYC, mycoprotein; PCM, protein concentrated from mycoprotein; 1RM, one repetition maximum.

No significant differences between groups.

### Pre-Testing

Once accepted to the study, all participants underwent a pre-testing protocol at least 5 d before a single experimental trial. Participants reported to the laboratory to assess body composition and unilateral leg strength and to become familiarised with the exercise protocol to be used during the experimental trial (described below). Body composition (body fat (%) and lean mass (kg)) was assessed using Air Displacement Plethysmography (BodPod, Life measurement, Inc.). Participants were also provided with a 3-d food diary (two weekdays and one weekend day) to assess habitual dietary intake.

### Experimental protocol

**Fig. 1.**
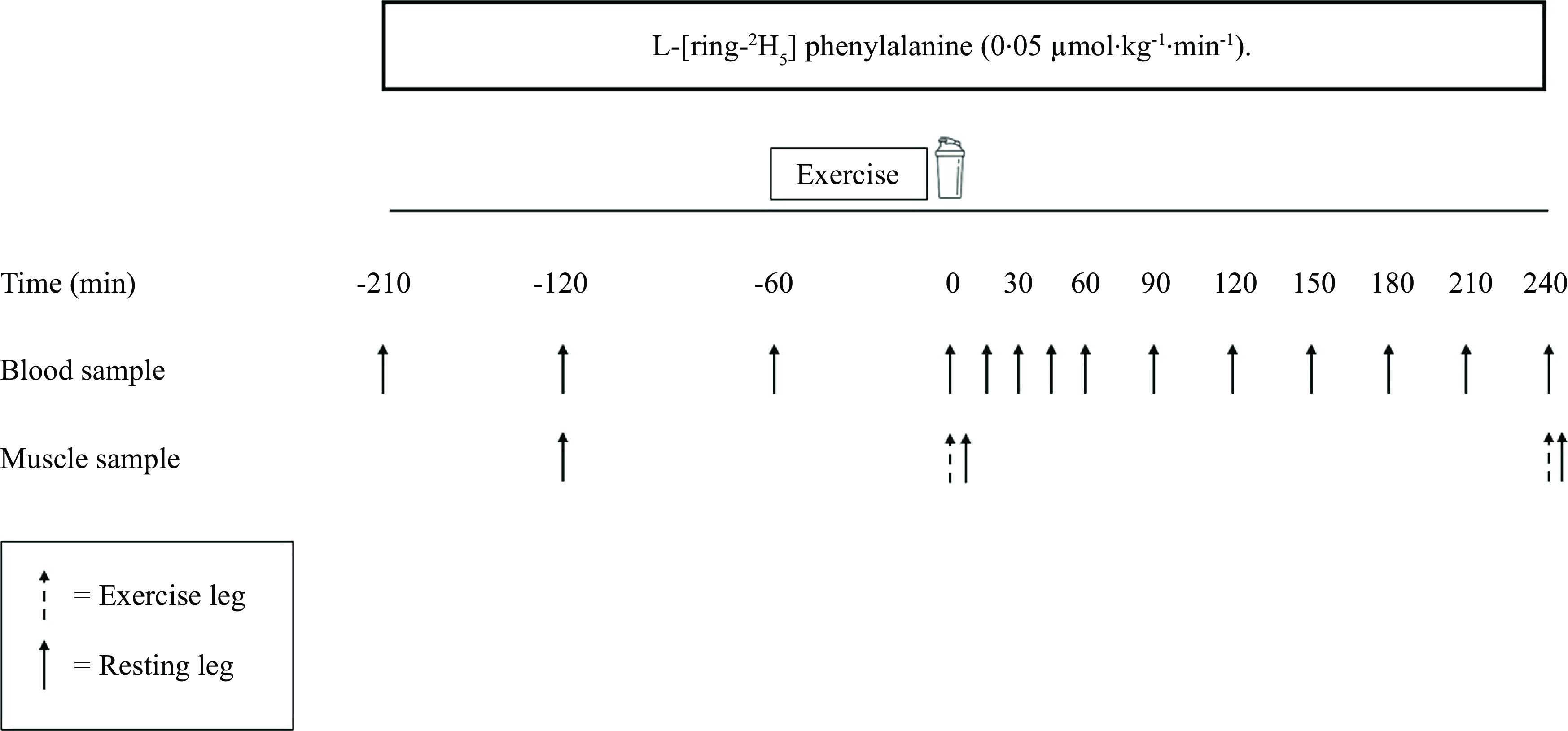
Protocol schematic of the experimental visit.

Participants were randomly assigned to one of two parallel groups and completed a single experimental trial in a double-blind manner. An overview of the experimental protocol can be found in Fig. 1. Participants were asked to avoid vigorous physical activity and alcohol for 48 h preceding the trial. All participants were provided with a standardised meal to consume as their last food intake 10 h before arriving at the laboratory (4·6 MJ (1110 kcal), 29 % energy from fat, 46 % energy from carbohydrate and 25 % energy from protein). On the day of the trial, participants arrived at the laboratory at 08.00 after a 10 h overnight fast. A Teflon^TM^ cannula was inserted into an antecubital vein of one arm for the infusion of the stable isotope tracer. Before the infusion was initiated, a baseline venous blood sample was taken from this site to measure background isotopic enrichments. Following the baseline blood sample, the infusion protocol began with a single intravenous priming dose of L-[*ring*-^2^H_5_]phenylalanine (2·12 μmol/kg) (*t* = –210 min). After the priming dose, a continuous tracer infusion was initiated (*t* = –210 min) at a rate of 0·05 μmol·kg^–1^·h^–1^ for L-[*ring*-^2^H_5_]phenylalanine for the duration of the protocol. Once this infusion was in progress, a second Teflon^TM^ cannula was inserted retrogradely into a dorsal hand vein of the contralateral arm and placed in a heated hand unit (55°C) to allow for subsequent arterialised venous blood sampling^(17)^. Following 90 min of continuous infusion (t = –120 min), arterialised venous blood samples were then taken throughout the remainder of the infusion at the following time (*t*) points: –120, –60, 0, 15, 30, 45, 60, 90, 120, 150, 180, 210 and 240 min. A baseline muscle biopsy sample was collected at *t* = –120 min from the non-dominant (resting) leg. Muscle biopsies were collected from the *m. vastus lateralis* (approximately 15 cm from the patella) with a modified Bergström suction needle under local anaesthesia (2 % lidocaine). All biopsy samples were immediately freed from any visible blood, connective and adipose tissue before being frozen in liquid N_2_ (within 30–60 s) and stored at –80°C until analysis. At –60 min, participants were taken by a wheelchair to the research gymnasium in order to execute a bout of unilateral leg resistance exercise, as described below. Participants were taken by a wheelchair back to the laboratory such that no weight bearing was performed with the resting leg throughout the experiment. Following exercise, bilateral muscle biopsies were collected from the rested (at least 1–2 cm distal from the baseline incision) and exercised legs (120 min after the initial biopsy; *t* = 0). Immediately following the biopsies (*t* = 0), participants consumed either a mycoprotein (MYC, 45 % protein, 10 % carbohydrate, 13 % fat, 25 % fibre) or protein concentrated from mycoprotein (PCM, 73 % protein, 0·1 % carbohydrate, 2 % fat and 18 % fibre) beverage (administered randomly in a double-blind fashion) within an allotted 5 min time period. Following beverage consumption, participants were asked to identify the protein beverage they consumed, with 67 % (11 from 12 in MYC and 5 from 12 in PCM) guessing correctly, implying partial success of the blinding procedures. Participants then rested in a semi-supine position for 4 h, after which another set of bilateral muscle biopsies were collected at least 2 cm distal to the previous incisions, completion of which indicated the end of the experiment after which participants were provided with food and transport home. Average incorporation time between the collection of biopsies for the calculation of postabsorptive MyoPS did not differ between groups in either rested (MYC, 128 ± 2 min; PCM, 126 ± 1 min; *P* > 0·05) or exercised legs (MYC, 123 ± 1 min; PCM 122 ± 1: *P* > 0·05). Average incorporation times between the collection of biopsies used to calculate postprandial MyoPS rates did not differ between groups in either rested (MYC, 245 ± 2 min; PCM 244 ± 2 min; *P* > 0·05) or exercised (MYC, 244 ± 1 min; PCM, 244 ± 1 min; *P* > 0·05) legs.

### Resistance exercise protocol

During the pre-testing visit, unilateral 3 repetition max (RM) was assessed to estimate 1RM for leg press and leg extension exercises^(18)^. The dominant leg (only) was used for the exercise throughout to maximise weight lifted, optimise movement execution and thereby create a maximal exercise stimulus. 3RM, rather than 1RM, was selected to predict 10RM to minimise safety risks. Strength testing began with a brief warm-up/practice of each exercise. Thereafter, participants attempted a self-selected weight for 3RM. Weight was increased for each subsequent attempt with final 3RM being accepted as the last weight lifted correctly before a failed attempt (±5 kg from failed attempt). 10RM was then calculated as 70 % of estimated 1RM. Once 3RM testing had finished, participants rested for roughly 5 min and were then asked to complete one set (10 repetitions) at the calculated 70 % 1RM for familiarisation and verification purposes.

For the experimental trial, participants completed a brief warm-up of leg press exercise consisting of ten repetitions at 50 % 1RM followed by eight repetitions at 60 % 1RM performed unilaterally with the dominant leg as per pre-testing. Thereafter, participants executed four unilateral sets of leg press followed by four sets of leg extension, each separated by 120 s rest. Sets were performed at 70 % 1RM, and participants were encouraged to work to volitional failure during each set, aiming to fail at 10 repetitions. For subsequent sets, the weight was increased when participants were able to perform > 12 repetitions and decreased when participants were unable to perform eight repetitions. Verbal encouragement was provided throughout, and the rested, non-dominant leg was kept relaxed and unloaded throughout.

### Protein beverage preparation

Freeze-dried mycoprotein and protein isolated from mycoprotein were produced and provided by Marlow Foods Ltd, Quorn Foods. Natural mycoprotein was produced as previously described^(19)^. Briefly, *Fusarium venenatum* for mycoprotein production was grown via continuous aerobic flow fermentation, with the addition of carbohydrate and ammonia substrates, under tightly controlled conditions (temperature 28–30°C and pH 6·0). The mycelium of the fungus is heat-treated (72–74°C for 30–45 min) to reduce RNA concentrations, then further heat-treated at 90°C. The suspended hyphae are then centrifuged with the resultant solid mass further concentrated by vacuum chilling. The produced mycoprotein was then milled and freeze-dried to produce a powder (45 % protein). For the production of the novel protein concentrate, 250 kg of frozen mycoprotein pads (DM 23·9 %; protein concentration 140 g/kg) was passed through a frozen meat grinder. This was then added to an agitator extraction tank with 7·4 litre of water + 0·167 litre of 20 % sodium hydroxide per kg of mycoprotein pad. This was then centrifuged, and any non-soluble material was removed. The remaining protein solution was then precipitated by the addition of sulphuric acid, reducing the pH to 3·5. The precipitated protein was then centrifuged, and the supernatant was removed. The resulting precipitate was suspended and washed in 1500 litre of water. This wash step was repeated before being centrifuged, and the water supernatant was removed. Following removal of the water, the pH was then adjusted to 5·5 by adding sodium hydroxide and the sample was then dried. Finally, the protein went through an ethanol wash and evaporation step to maximise the removal of residual lipids, producing 8·1 kg of protein concentrate powder (73 % protein). Both protein sources were independently analysed (Premier Analytical Services) for energy, macronutrient content and amino acid composition, the details of which are displayed in Table 2.

**Table 2.** The nutritional composition of the experimental beverages (70 g and 38·2 g of mycoprotein and protein concentrated from mycoprotein, respectively)

	MYC	PCM
Macronutrients		
Protein (g)	31·5	28·0
Carbohydrate (g)	7	0
Fat (g)	9	0·6
Fibre (g)	17·5	7·0
Energy (kcal)	238	132
Energy (kJ)	996	558
Amino acid content (g)		
Alanine	2·0	1·8
Arginine	2·2	2·0
Asparagine	3·3	3·0
Cysteine	0·2	0·2
Glutamine	3·9	3·7
Glycine	1·5	1·4
Histidine	0·8	0·7
Isoleucine	1·5	1·6
Leucine	2·5	2·5
Lysine	2·6	2·4
Methionine	0·4	0·5
Phenylalanine	1·5	1·4
Proline	1·6	1·3
Serine	1·6	1·4
Threonine	1·7	1·6
Tryptophan	0·4	0·4
Tyrosine	1·2	1·2
Valine	1·9	1·9

MYC, mycoprotein; PCM, protein concentrated from mycoprotein.

Protein content (g) is calculated from the sum of the amino acids measured after complete hydrolysis.

The evening before the experimental trial, the powdered protein sources were assimilated with 400 ml of water and 20 ml of energy-free flavouring (Clearwater), blended for roughly 2 min and refrigerated overnight (420 ml final volume). Drinks were enriched (2 %) with L-[*ring*-^2^H_5_]phenylalanine to maintain systemic isotopic steady state following protein ingestion^(12)^. During the experimental trial, once participants had consumed the drink, an additional 100 ml of water was added to ‘rinse’ the bottle and ensure that all the protein had been consumed. All drinks were well tolerated and consumed within the allotted 5 min (average time to consume was 3·7 ± 0·5 and 3·6 ± 0·6 min for MYC and PCM, respectively; *P* > 0·05) with no adverse effects reported during the test day or on follow-up calls on subsequent days. Once consumed, participants were asked to subjectively rate the drink from 1 (bad) to 10 (good) for tolerability (MYC, 4·2 ± 0·5; PCM, 5·0 ± 0·5; *P* > 0·05). Double-blinding of the drinks was achieved by having a separate researcher from those carrying out the experimental trial visits prepare the drinks in an opaque bottle. The drinks were matched for leucine content (2·5 g) requiring 70 and 38·2 g of powder, thereby providing 31·4 and 28·0 g of protein in MYC and PCM, respectively.

### Blood sample collection and analyses

Ten millilitre of arterialised venous (with the exception of the baseline sample which was a venous collection) blood was collected into a syringe at each time point. Five millilitre of that sample was added to EDTA-containing tubes (BD vacutainer LH; BD Diagnostics, Nu-Care) and centrifuged for 10 min at 4000 rpm at 4°C. The plasma supernatant was then removed, aliquoted and stored at −80°C for later analyses. The remaining 5 ml of blood was added to additional vacutainers (BD vacutainers SST II, BD Diagnostics, Nu-Care) and left upright to clot at room temperature for 30 min and then centrifuged for 10 min at 4000 rpm at 4°C. The serum supernatant was then removed, aliquoted and stored at −80°C for future analyses.

Serum insulin concentrations were measured using a commercially available ELISA kit (DRG Insulin ELISA, EIA-2935, DRG International Inc.). Plasma L-[*ring*-^2^H_5_]phenylalanine enrichments (tracer/tracee ratio and mole percent excess) and concentrations of phenylalanine, leucine, valine, isoleucine, lysine, histidine, glutamic acid, methionine, proline, serine, threonine, tyrosine and alanine were determined in tert-butyldimethylsilyl derivatives by GC-MS with electron impact ionisation (Agilent) as described previously^(20)^. Briefly, to prepare samples for GC-MS, 10 μl of 2 mM norleucine was added as an internal standard to 450 μl of plasma and deproteinised on ice with 450 μl of 15 % 5-sulfosalcylic acid. Samples were then vortexed and centrifuged at 4000 rpm for 10 min at 4°C. The supernatant was then loaded onto cation-exchange columns. Columns were then filled with ddH_2_O, followed by 6 ml of 0·5 M acetic acid and then washed once more with ddH_2_O, with the columns allowed to drain between each step. The amino acids were then eluted with 2 ml of 6 M ammonia hydroxide (NH_4_OH). The eluate was dried using a Speed-Vac for 8 h at 60°C.

### Muscle tissue analyses

Myofibrillar protein extractions were performed as previously described^(21)^. The process was carried out with approximately 50 mg of muscle tissue, which was homogenised using a mechanical glass pestle in a glass tube in a homogenisation buffer (in mM: 50 TRIS·HCl pH 7·4, 1 EDTA, 1 EGTA, 10 *β*-glycerophosphate salt, 50 NaF and 0·5 activated Na3VO4 (Sigma-Aldrich Company Ltd)) with a complete protease inhibitor cocktail tablet (1 tablet per 50 ml of buffer, Roche). The homogenate was transferred into a clean 2 ml Eppendorf and centrifuged at 2200 *g* for 10 min at 4°C. The supernatant (sarcoplasmic fraction) was aliquoted and stored at –80°C for subsequent western blot analysis. The remaining pellet was then washed in 500 μl of homogenisation buffer and centrifuged again at 700 *g* for 10 min at 4°C, and the resultant supernatant was discarded. The remaining protein portion (myofibrillar and collagen)^(22)^ was then solubilised in 750 μl of 0·3 M sodium hydroxide and heated at 50°C for 30 min and centrifuged at 10 000 *g* for 5 min at 4°C. The supernatant (myofibrillar fraction) was then aliquoted into a new 2 ml Eppendorf and precipitated in 500 μl of 1 M perchloric acid. These samples were centrifuged at 700 *g* for 10 min at 4°C, and the resultant supernatant was discarded. The remaining myofibrillar pellet was washed in 1 ml of 70 % ethanol and centrifuged at 700 *g* for 5 min at 4°C before the ethanol was removed. This step was repeated once more before the amino acids were then hydrolysed by adding 2 ml of 6 M hydrochloric acid and heating at 110°C for 24 h. Once hydrolysed, the amino acids were then dried using a Speed-Vac for 4 h at 80°C. Samples were then reconstituted in 1·5 ml of 25 % acetic acid and pipetted into the cation-exchange column. The Eppendorf was then rinsed with another 1·5 ml of 25 % acetic acid. The columns were then eluted with 2 ml of 6 M NH_4_OH into a 2 ml Eppendorf, and the eluate was dried in a Speed-Vac for 8 h at 60°C. Samples were cleaned by adding 1 ml of ddH_2_O and 1 ml of 0·1 % formic acid in acetonitrile and centrifuged at 10 000 *g* for 3 min at 4°C. The supernatant was aliquoted into a new Eppendorf and dried in the Speed-Vac for 5 h at 60°C. In order to derivatise the muscle sample, 50 μl of MTBSTFA + 1 % tertbutyl-dimethylchlorosilane and 50 μl of acetonitrile were added to the dry samples, vortexed and heated at 95°C for 45 min^(23)^. The samples were analysed by GC-MS (7890 GC coupled with a 5975 MSD, Agilent Technologies) in triplicate using electron impact ionisation and selected ion monitoring for the measurement of isotope ratios^(24)^. One microlitre of the sample was injected in splitless mode (injector temperature: 280°C). Peaks were resolved using an HP5-MS 30 m × 0·25 mm ID × 0·25 μm capillary column (Agilent). He was used as the carrier gas at 1·2 ml/min constant flow rate. The temperature ramp was set from 80–245°C at 11°C/min, then to 280°C at 40°C/min^(24)^. Selected ion recording conditions were used to monitor fragments m/z 237 and 239, respectively, for the m + 3 and m + 5 fragments of phenylalanine-bound protein and m/z 336 and 341, respectively, for the m + 0 and m + 5 fragments of the phenylalanine-free fraction. A single linear standard curve from mixtures of known m + 5/m + 0 ratios for L-[*ring*-^2^H_5_]phenylalanine was used to determine the enrichments of the protein-bound samples using the m + 5/m + 3 ratio.

The sarcoplasmic fractions obtained from the myofibrillar amino acid extractions were analysed for total and phosphorylated mammalian target of rapamycin (mTOR and p-mTOR Ser^2448^, respectively) contents as an indirect measure of mTOR activity. Total protein content of the sarcoplasmic fraction was measured using a colorimetric assay (DC protein assay, Bio-Rad Laboratories, Inc.). Proteins were unfolded at 95°C in XT sample buffer (Bio-Rad Laboratories, Inc.), and 20 μg of protein was then loaded per lane in duplicate onto a 3–8 % TRIS acetate polyacrylamide gel and separated by electrophoresis in XT tricine running buffer for 65 min at 150 V. Proteins were transferred to a 0·2 μM nitrocellulose membrane using Trans-blot turbo transfer system (Bio-Rad Laboratories, Inc.) at 2·5 A and 25 V for 10 min. Membranes were blocked in 5 % bovine serum albumin in TRIS-buffered saline, 0·1 % Tween (TBST) for 1 h, before incubation in rabbit anti-phospho-mTOR Ser^2448^ monoclonal antibody (5536, Cell Signaling Technology, Inc.; 1:1000 in TBST) and rabbit anti-*α*-tubulin (11H10, Cell Signaling Technology, Inc.; 1:20 000 in TBST) loading control overnight at 4°C. Membranes were washed (3 × 10 min) in TBST before 1 h incubation in secondary horseradish peroxidase-conjugated anti-rabbit IgG antibody (ab6721, Abcam PLC; 1:3000 in TBST) at room temperature. Following another wash (3 × 10 min) in TBST, membranes were submerged in Clarity Western chemiluminescent detector solution for 5 min (Bio-Rad Laboratories, Inc.). The membranes were then imaged using a Chemidoc scanner (Bio-Rad Laboratories, Inc.), and band intensities were quantified using Image Lab software (Bio-Rad Laboratories, Inc.). The expected migration of p-mTOR (289 kDa) and *α*-tubulin (52 kDa) was confirmed using a kaleidoscope protein ladder (Bio-Rad Laboratories, Inc.). For total mTOR, the membranes were incubated in stripping buffer for 30 min at room temperature (Thermo Fisher Scientific), blocked for 1 h in 5 % bovine serum albumin in TBST and re-probed overnight with an anti-mTOR monoclonal primary antibody (2972, Cell Signaling Technology, Inc.; 1:1000 in TBST) and anti-*α*-tubulin at 4°C, and the above steps were repeated to obtain corresponding bands for total mTOR. The band densities of both p-mTOR and total mTOR were calculated as a ratio against the *α*-tubulin band within each lane, and p-mTOR was calculated as a ratio of total mTOR (i.e. indicating ‘phosphorylation status’). Fold change in p-mTOR was calculated from basal to fed conditions in rested and exercised legs.

### Calculations

The fractional synthetic rates (FSR) of myofibrillar proteins were calculated using the standard precursor-product equation^(20)^:

*FSR (%·h–1) = (ΔEp × t/ Eprecursor × t) × 100*

where *ΔEp* is the increment in protein-bound L-[*ring*-^2^H_5_]phenylalanine in myofibrillar protein between two muscle biopsies, *E*_precursor_ is the average L-[*ring*-^2^H_5_]phenylalanine enrichment in the plasma precursor pool over time and *t* indicates the time (*h*) between two muscle biopsies.

### Statistical analysis

A two-sided power analysis with expected effect sizes estimated from previous research^(12,25)^ revealed that ten in each group were sufficient to detect expected differences in postprandial MyoPS rates between protein conditions (MYC *v*. PCM) when using a repeated-measures ANOVA (*P* < 0·05, power 80 %, f = 0·67; G × power version 3.1.9.2). Factoring in a 20 % dropout rate, twenty-four participants were therefore recruited for the study. Statistical significance was set at *P* < 0·05. All calculations were performed on GraphPad 7.1. Participants’ characteristics, total work done and background L-[*ring*-^2^H_5_]phenylalanine enrichments were analysed using independent-samples *t* tests. Differences in serum insulin concentrations, plasma amino acid concentrations, plasma tracer enrichments and myofibrillar L-[*ring*-^2^H_5_]phenylalanine enrichments were compared using two-way (group (MYC *v*. PCM) × time) repeated-measures ANOVA. Separate two-way ANOVA was performed on fasted and fed plasma L-[*ring*-^2^H_5_]phenylalanine enrichments. Myofibrillar FSR were analysed using a three-way (group *×* time *×* exercise) ANOVA. Total postprandial amino acid availability was calculated as incremental AUC (iAUC) with baseline set as *t* = 0.

## Results

### Participants’ characteristics

No differences in body mass, height, BMI, body fat percentage, lean mass or leg strength (1RM) were found between groups (all *P* > 0·05; see Table 1). Total work performed (repetition *×* load) during the experimental exercise bout did not differ between groups for leg press (MYC, 4401 ± 227 kg; PCM, 4792 ± 325 kg), leg extension (MYC, 1486 ± 97 kg; PCM, 1573 ± 86 kg) or across the full exercise protocol (MYC; 5887 ± 296 kg; PCM; 6366 ± 369 kg) (all *P* > 0·05).

### Serum insulin and plasma amino acid concentrations

**Fig. 2.**
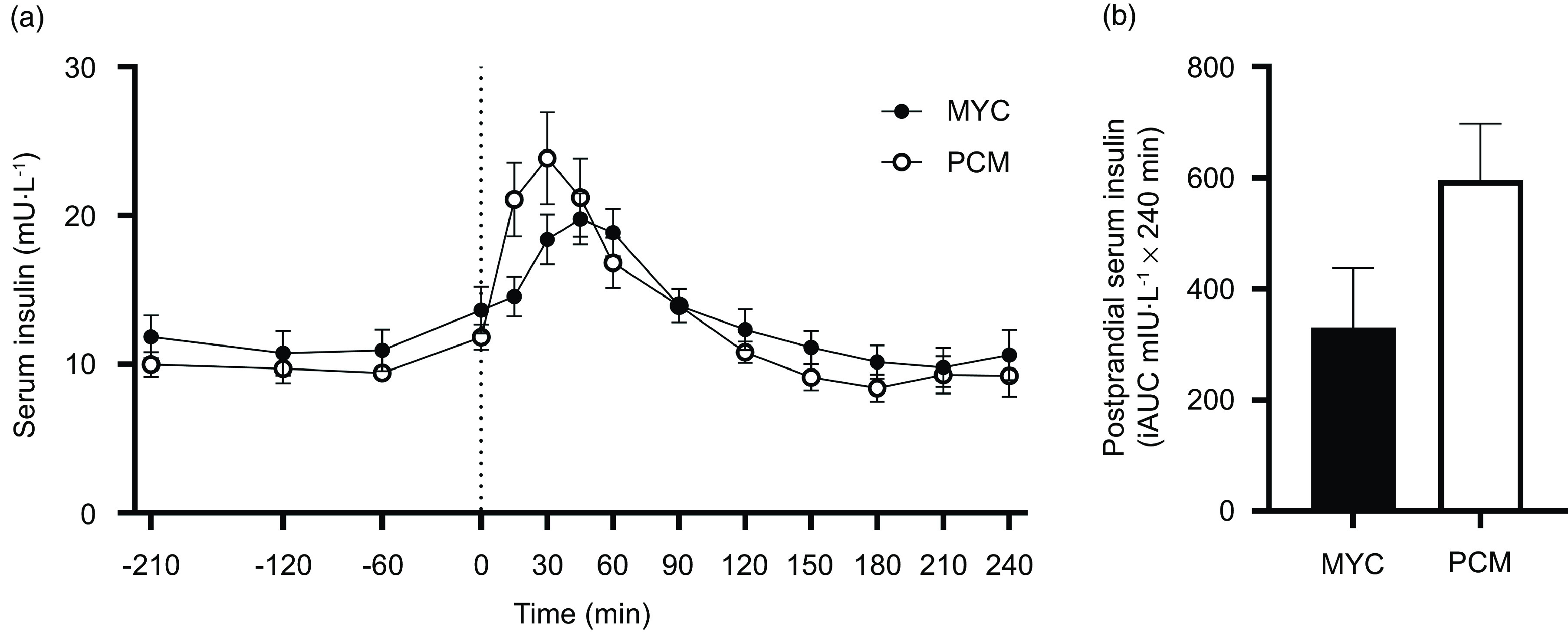
Time course (a) and incremental AUC (iAUC; calculated as above postaborptive values) (b) of serum insulin concentrations for a 3·5 h postabsorptive period (time course only) and a 4 h postprandial period in healthy resistance-trained men. The dashed vertical line represents drink consumption (70 g of mycoprotein containing 31·5 g protein and 2·5 g leucine (MYC; *n* 12) or 38·2 g of protein concentrated from mycoprotein containing 28·0 g protein and 2·5 g leucine (PCM; *n* 12)), and execution of a bout of unilateral resistance leg exercise. Time course data were analysed using a two-way repeated-measures ANOVA (group × time) with Sidak *post hoc* tests used to detect differences at individual time points. iAUC data were analysed using an independent-samples *t* test. Time × group interaction; *P* < 0·001. Values are mean ± sem.

All serum insulin and plasma amino acid concentration data are presented for twenty-four males (twelve MYC and twelve PCM). Serum insulin concentrations over the time course of the experiment are depicted in Fig. 2. Fasting serum insulin concentrations were similar between groups (MYC, 12 ± 5 mU·l^−1^; PCM, 10 ± 3 mU·l^−1^). The ingestion of protein significantly increased fasting serum insulin concentrations (time effect; *P* < 0·0001) to a different extent between groups (time × group interaction; *P* < 0·001). Postprandial serum insulin concentrations in the MYC group were elevated compared with baseline from 30 to 60 min (*P* < 0·05), peaked at 45 min (20 ± 6 mU·l^−1^) and had returned to fasting levels by 90 min. In the PCM group, postprandial insulin concentrations rose more rapidly (greater than baseline from 15 to 60 min; *P* < 0·05) peaked earlier (30 min; at 24 ± 11 mU·l^−1^) but also returned to fasting concentrations by 90 min. Despite these divergent temporal responses, serum insulin concentrations did not differ between groups at any specific time point. There was a trend for greater postprandial serum insulin iAUC in the PCM compared with MYC group (*P* = 0·085).

**Fig. 3.**
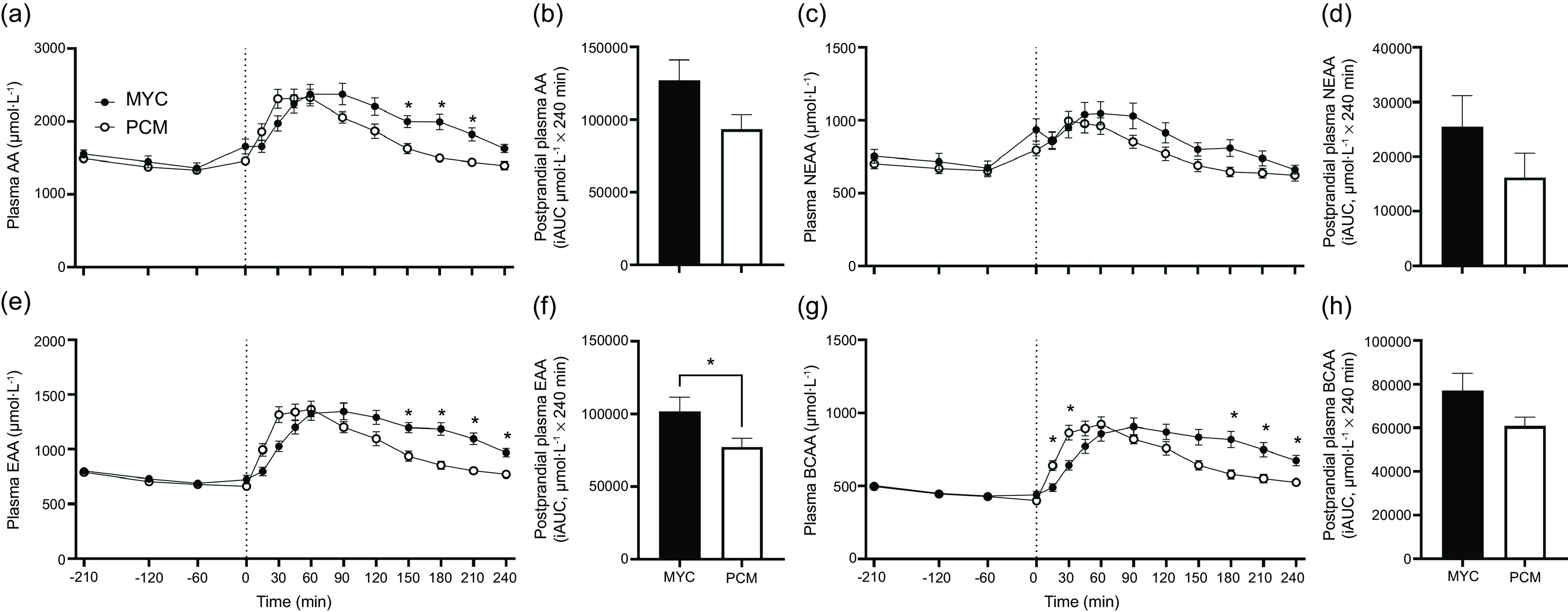
Time course and incremental AUC (iAUC; calculated as above postaborptive values) of amino acids (AA) (a), (b), non-essential amino acids (NEAA) (c), (d), essential amino acids (EAA) (e), (f), and branch chain amino acids (BCAA) (g), (h) over a 3·5 h postabsorptive period (time course only) and 4 h postprandial period in healthy resistance-trained men. The dashed vertical line represents drink consumption (70 g of mycoprotein containing 31·5 g protein and 2·5 g leucine (MYC; *n* 12) or 38·2 g of protein concentrated from mycoprotein containing 28·0 g protein and 2·5 g leucine (PCM; *n* 12)), and execution of a bout of unilateral resistance leg exercise. Time course data were analysed using a two-way repeated-measures ANOVA (group × time) with Sidak *post hoc* tests used to detect differences at individual time points. iAUC data were analysed using an independent-samples *t* test. *Individual differences between conditions at that time point and a difference between conditions on the bar graphs (*P* < 0·05). Time × group interaction; all *P* < 0·001. Values are mean ± sem.

**Fig. 4.**
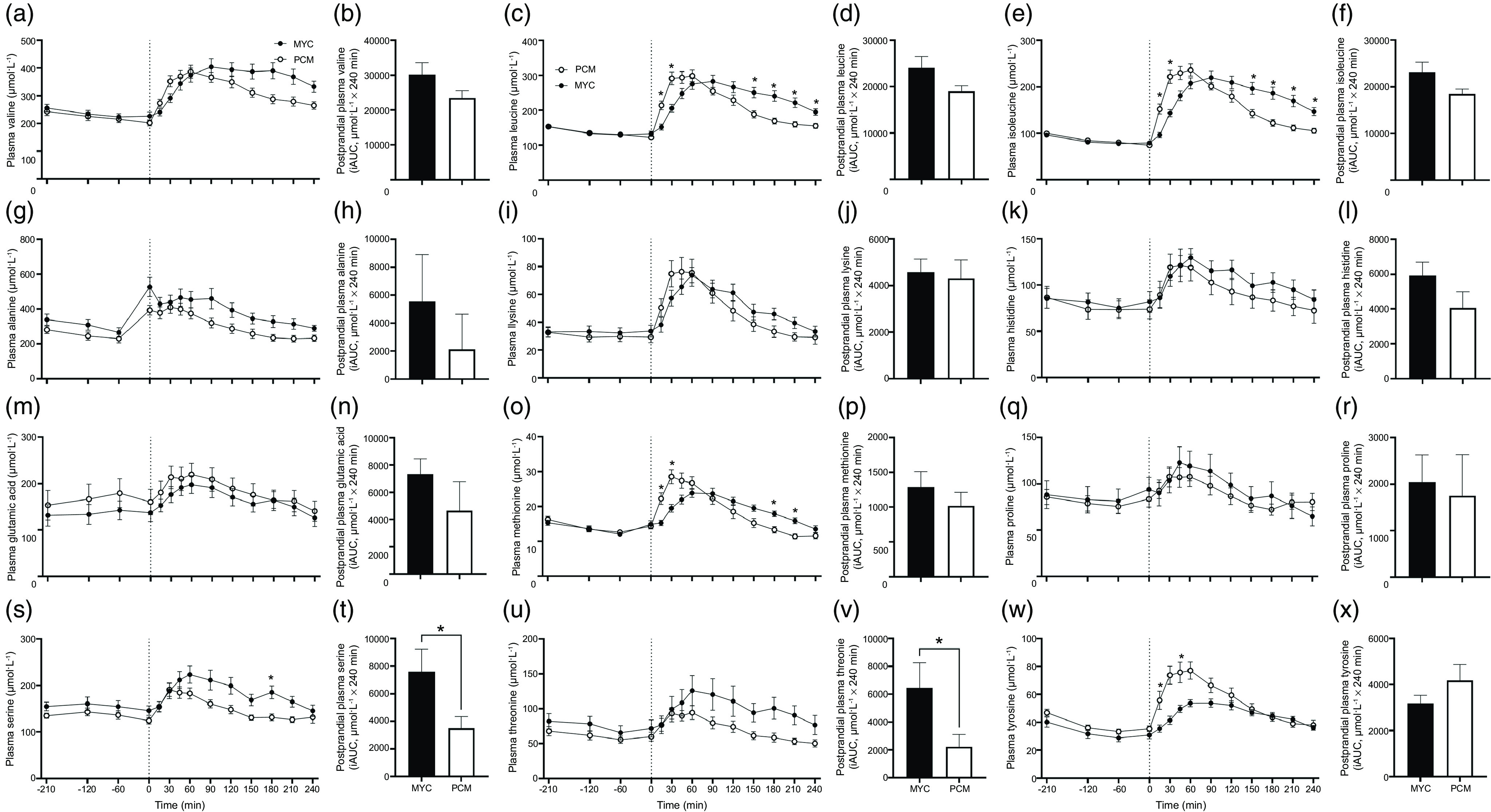
Time course and incremental AUC (iAUC; calculated as above postaborptive values) of valine (a), (b), leucine (c), (d), isoleucine (e), (f), alanine (g), (h), lysine (i), (j), histidine (k), (l), glutamic acid (m), (n), methionine (o), (p), proline (q), (r), serine (s), (t), threonine (u), (v), and tyrosine (w), (x) over a 3·5 h postabsorptive period (time course only) and 4 h postprandial period in healthy resistance-trained men. The dashed vertical line represents drink consumption (70 g of mycoprotein containing 31·5 g protein and 2·5 g leucine (MYC; *n* 12) or 38·2 g of protein concentrated from mycoprotein containing 28·0 g protein and 2·5 g leucine (PCM; *n* 12)), and execution of a bout of unilateral resistance leg exercise. Time course data were analysed using a two-way repeated-measures ANOVA (group × time) with Sidak *post hoc* tests used to detect differences at individual time points. iAUC data were analysed using an independent-samples *t* test. *Individual differences between conditions at that time point and a difference between conditions on the bar graphs (*P* < 0·05). Time × group interaction (all *P* < 0·001) except glutamic acid and proline (both *P* > 0·05). Values are mean ± sem.

Plasma total amino acid, essential amino acid, branched-chain amino acid, non-essential amino acid and individual amino acid concentrations over the time course of the experiment are presented in Fig. 3 and 4. All plasma amino acid concentrations increased following protein ingestion (time effects; all *P* < 0·0001). Plasma concentrations of total amino acid, non-essential amino acid, essential amino acid and branched-chain amino acid all displayed a more rapid increase following protein ingestion in the PCM compared with MYC group (time × group interactions; all *P* < 0·0001). Following protein ingestion, plasma total amino acid concentrations remained elevated for longer in the MYC compared with PCM group with higher concentrations observed at 150, 180 and 210 min (all *P* < 0·05). Similar responses were also observed for plasma essential amino acid and branched-chain amino acid concentrations, with significantly higher concentrations observed post-protein ingestion between 150 and 240 min (*P* < 0·05) and 180 and 240 min (*P* < 0·05), respectively, in MYC compared with PCM. Plasma leucine and isoleucine concentrations increased more rapidly in the PCM group (time *×* group interaction; *P* < 0·0001) with greater concentrations detected at 15 and 30 min compared with the MYC group (*P* < 0·05). Plasma leucine and isoleucine concentrations remained elevated following protein ingestion for longer in the MYC compared with PCM group with greater concentrations observed between 150 and 240 min (*P* < 0·05). Aside from glutamic acid and proline (time *×* group interaction, both *P* > 0·05), all individual plasma amino acid and non-essential amino acid concentrations increased to differing degrees dependent on the protein source ingested (time *×* group interactions; all *P* < 0·001).

Total postprandial plasma amino acid availabilities, as indicated by iAUC, are also displayed in Fig. 3 and 4 for all amino acid parameters (inset graphs). Greater postprandial plasma availability was observed in the MYC compared with PCM group for essential amino acid, serine and threonine (all *P* < 0·05), and trends (all *P* < 0·1) observed for total amino acid, branched-chain amino acid, leucine and isoleucine. Postprandial plasma availability for all other amino acid parameters did not differ between groups (all *P* > 0·05).

### Plasma and skeletal muscle tracer analysis

The samples of two participants from the PCM group were excluded from tracer analyses due to insufficient muscle tissue. Therefore, all data for plasma and muscle L-[*ring*-^2^H_5_]phenylalanine are for twenty-two males (twelve MYC and ten PCM).

**Fig. 5.**
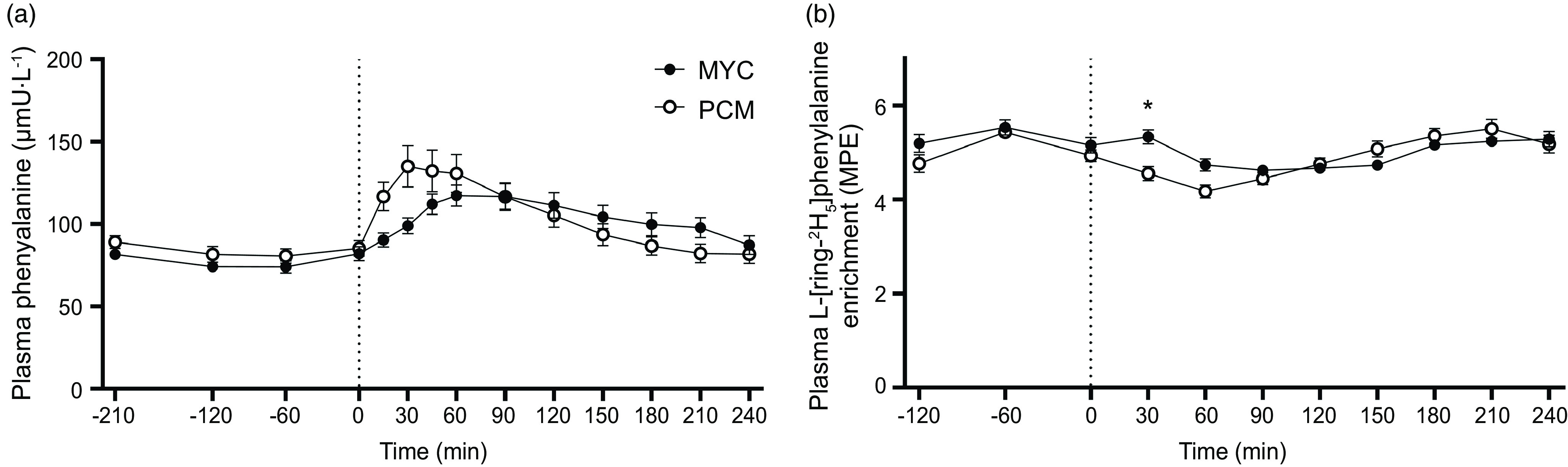
Time course of plasma phenylalanine concentrations (a) and plasma L-[ring-^2^H_5_]phenylalanine enrichments (b) during the experimental trial over a 3·5 h postabsorptive period and 4 h postprandial period in healthy resistance-trained men. The dashed vertical line represents drink consumption (70 g of mycoprotein containing 31·5 g protein and 2·5 g leucine (MYC; *n* 12) or 38·2 g of protein concentrated from mycoprotein containing 28·0 g protein and 2·5 g leucine (PCM; *n* 12)), and execution of a bout of unilateral resistance leg exercise. Time course data were analysed using a two-way repeated-measures ANOVA (group × time) with Sidak *post hoc* tests used to detect differences at individual time points. iAUC data were analysed using an independent-samples *t* test. *Individual differences between conditions at that time point and a difference between conditions on the bar graphs (*P* < 0·05). Time × group interaction; all *P* < 0·001. Values are mean ± sem. MPE, mole percent excess.

The time course of plasma L-[*ring*-^2^H_5_]phenylalanine enrichments is displayed in Fig. 5. Plasma L-[*ring*-^2^H_5_]phenylalanine enrichments changed over time (time effect; *P* < 0·05), but to the same extent between groups (*P* > 0·05) in the postabsorptive period (time *×* group interaction; *P* > 0·05). Plasma L-[*ring*-^2^H_5_]phenylalanine enrichments decreased transiently (between 60 and 120 min, and between 30 and 90 min in MYC and PCM, respectively) following protein ingestion in both groups (time effect; *P* < 0·0001) and more rapidly so in PCM compared with the MYC group (time × group interaction; *P* < 0·0001). By 120 min, a steady state had been regained in both groups.

Myofibrillar protein-bound L-[*ring*-^2^H_5_]phenylalanine enrichments were equivalent between groups at baseline (*P* > 0·05). Myofibrillar protein-bound L-[*ring*-^2^H_5_]phenylalanine enrichments increased but to the same extent in both groups (time effect; *P* < 0·0001, time *×* group interaction; *P* > 0·05) during the postabsorptive period in the resting (from 0·0028 ± 0·0003 to 0·0062 ± 0·0008 in MYC and from 0·0035 ± 0·0007 to 0·0068 ± 0·0014 in PCM) and exercised (from 0·0028 ± 0·0003 to 0·0057 ± 0·0006 in MYC and from 0·0035 ± 0·0007 to 0·0059 ± 0·0009 in PCM) legs, with no differences between legs. Protein ingestion increased myofibrillar protein-bound L-[*ring*-^2^H_5_]phenylalanine enrichments to the same extent between groups (time effect; *P* < 0·0001, time × group interaction; *P* > 0·05) in the resting (from 0·0062 ± 0·0008 to 0·0186 ± 0·0017 in MYC and from 0·0068 ± 0·0014 to 0·0172 ± 0·0021 in PCM; *P* > 0·05) and exercised leg (from 0·0057 ± 0·0006 to 0·0221 ± 0·0015 in MYC and from 0·0059 ± 0·0009 to 0·0216 ± 0·0021 in PCM; time effect; *P* > 0·05) but to a greater extent in the exercised leg compared with the rested leg (exercise *×* time interaction; *P* < 0·01).

**Fig. 6.**
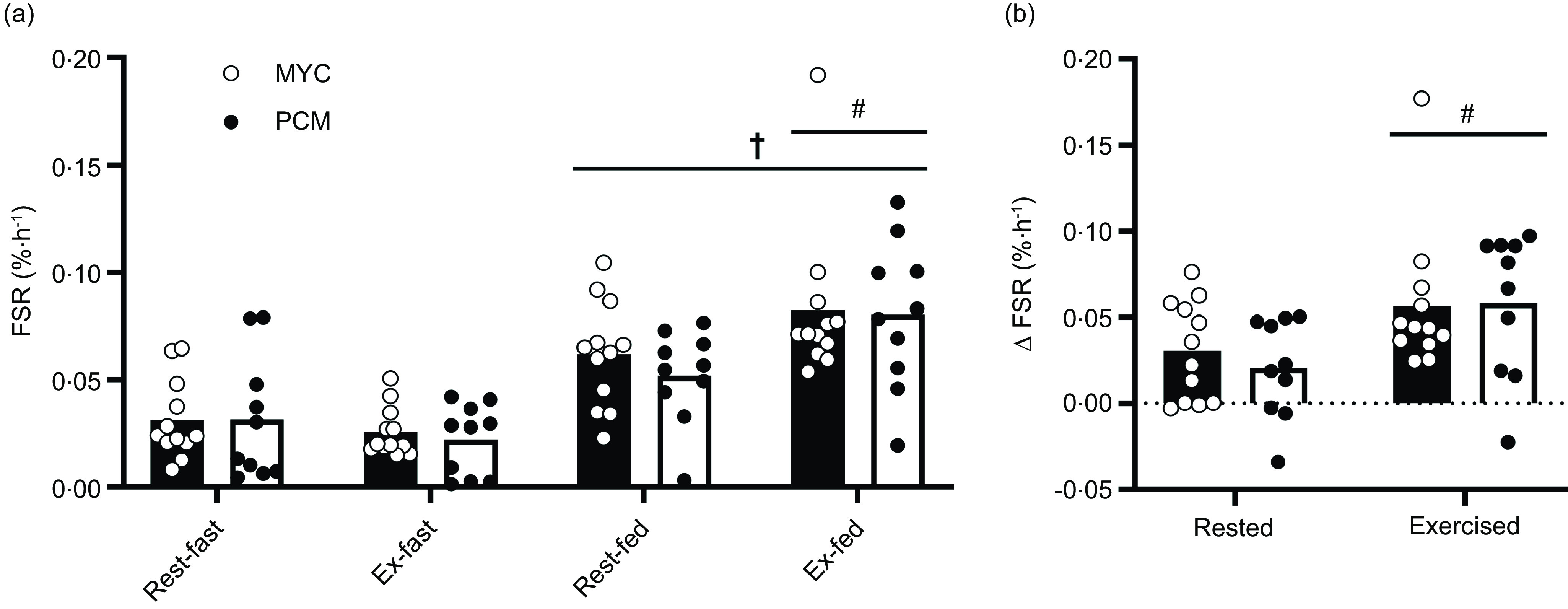
Myofibrillar protein fractional synthetic rates (FSR) calculated using the plasma L-[ring-^2^H_5_]phenylalanine precursor pool for a postabsorptive (fasted) and postprandial (fed) period (a) and delta FSR change from postabsorptive to postprandial state (b) for both MYC and PCM conditions in rested and exercised (unilateral leg press and leg extension) muscle in health young resistance-trained males. Postprandial state represents a 4 h period following drink consumption (70 g of mycoprotein containing 31·5 g protein and 2·5 g leucine (MYC; *n* 12) or 38·2 g of protein concentrated from mycoprotein containing 28·0 g protein and 2·5 g leucine (PCM; *n* 10)), and execution of a bout of unilateral resistance leg exercise. Data were analysed using a three-way (protein ingestion × group × exercise) ANOVA. Delta FSR data were analysed using a two-way (group × exercise) ANOVA. †Significant difference between fasting and fed conditions (main effect of protein ingestion; *P* < 0·0001). #A significant difference between rested and exercised tissue in fed conditions (exercise × feeding interaction; *P* < 0·01). Values are mean ± sem.

Myofibrillar FSR were calculated using the average plasma L-[*ring*-^2^H_5_]phenylalanine enrichments during the prandial period of interest as the precursor pool (Fig. 6). Postabsorptive myofibrillar FSR were similar between groups in both rested (MYC, 0·031 ± 0·005 %·h^−1^ and PCM, 0·031 ± 0·008 %·h^−1^; *P* > 0·05) and exercised (MYC, 0·026 ± 0·003 %·h^−1^ and PCM, 0·022 ± 0·004 %·h^−1^; *P* > 0·05) muscle, with no difference between legs. Protein ingestion increased myofibrillar FSR in rested and exercised muscle in both groups (time effect; *P* < 0·0001) but to a greater extent in exercised compared with rested tissue (exercise × time interaction; *P* < 0·01). The increase in myofibrillar FSR following protein ingestion was equivalent in both groups in rested and exercised tissue (group × time interaction and group × time × exercise interaction; *P* > 0·05). Myofibrillar FSR increased from 0·031 ± 0·008 to 0·062 ± 0·007 %·h^−1^ and from 0·026 ± 0·003 to 0·082 ± 0·010 %·h^−1^ in rested and exercised tissue, respectively, in the MYC group, and from 0·031 ± 0·009 to 0·052 ± 0·006 %·h^−1^ and 0·022 ± 0·005 to 0·080 ± 0·010 %·h^−1^ in rested and exercised tissue, respectively, in the PCM group. This also meant that the delta increases from postabsorptive to postprandial myofibrillar FSR were equivalent between groups in both rested (MYC, Δ0·031 ± 0·007 %·h^−1^ and PCM, Δ0·020 ± 0·008 %·h^−1^) and exercised (MYC, Δ0·057 ± 0·011 %·h^−1^ and PCM, Δ0·058 ± 0·012 %·h^−1^) muscle (group effect; *P* > 0·05), and the values were greater in the exercised compared with rested leg (exercise effect; *P* < 0·01).

### Myocellular signalling responses

**Fig. 7.**
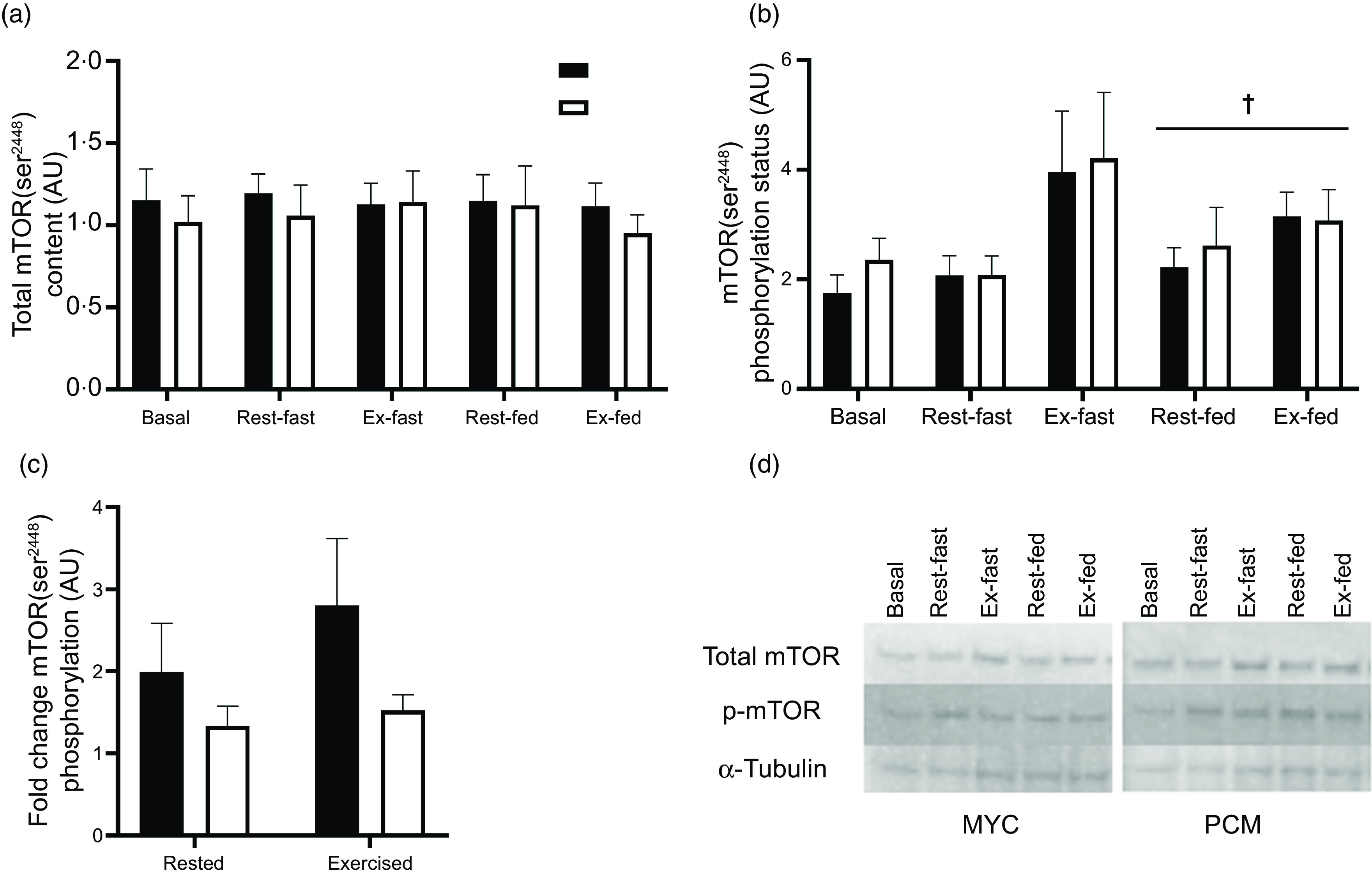
Total skeletal muscle mTOR content (**a**), muscle mTOR phosphorylation status (as a ratio of phosphorylated to total protein) (**b**), mTOR phosphorylation status (as a ratio of phosphorylated to total protein) fold change from basal to postprandial conditions in rested and exercised muscle tissue (**c**), and representative blots (**d**) for both groups. Basal bars represent total and phosphorylation status of mTOR before exercise and protein ingestion. Fasted bars represent total and phosphorylation status of mTOR immediately following exercise in the rested (rested fasted) and exercised (exercise fasted) legs. Fasted data points were excluded from the statistical analysis as the primary interest in the study is investigating change from basal to fed conditions. Postprandial bars (rested fed and exercise fed) represent total and phosphorylation status of mTOR 4 h following drink consumption (70 g of mycoprotein containing 31·5 g protein and 2·5 g leucine (MYC; *n* 12) or 38·2 g of protein concentrated from mycoprotein containing 28·0 g protein and 2·5 g leucine (PCM; *n* 12)), and execution of a bout of unilateral resistance leg exercise. Data were analysed using a three-way (time × group × exercise) ANOVA. Fold change data were analysed using a two-way ANOVA (group × exercise). All interactions and main effects for total skeletal muscle mTOR content (*P* > 0·05). Significant effect of feeding on mTOR phosphorylation status (time effect *P* < 0·05). All interactions and main effects for mTOR phosphorylation status fold change (*P* > 0·05). † A significant difference from basal condition (*P* < 0·01). Values are mean ± sem. mTOR, mammalian target of a rapamycin.

Myocellular signalling responses (Fig. 7) were determined in twenty-four males. Basal muscle total mTOR content and mTOR phosphorylation status did not differ between conditions (*P* > 0·05). Total mTOR content did not change in either group throughout the experiment nor differ between legs (time, exercise and group × time interaction; *P* > 0·05) (Fig. 7(a)). A three-way ANOVA of muscle mTOR phosphorylation status revealed a significant effect of time (*P* < 0·05) and exercise (*P* < 0·05) but did not detect a significant effect of group (*P* > 0·05) (Fig. 7(b)). However, the time effect observed here is likely driven by the high mTOR phosphorylation status in the exercised leg immediately after exercise and therefore does not represent an effect of protein ingestion. Given the effect this time point has on the statistical analysis, and that the primary interest of this experiment is to look at the change from basal to postprandial conditions, this time point (rested fasted and exercise fasted) has been excluded from further statistical analysis. Therefore, all muscle mTOR phosphorylation status data have subsequently been analysed from basal to fed (rested and exercise) conditions within a three-way ANOVA. Muscle mTOR phosphorylation status increased following protein ingestion (time effect; *P* < 0·05); however, the postprandial increase in mTOR phosphorylation did not differ between groups (group × time interaction; *P* > 0·05) or between rested and exercised muscle (exercise × time interaction; *P* > 0·05). There was a trend for differences in mTOR phosphorylation status fold change (from basal to fed state) between groups (group effect; *P* = 0·072) (Fig. 7(c)). However, there was no difference in fold change between rested and exercised legs (exercise effect; *P* > 0·05).

## Discussion

In the present work, we compared the MyoPS response to the ingestion of leucine-matched boluses of mycoprotein and a protein concentrated from mycoprotein in resting and exercised muscle tissue of young men. We hypothesised that the wholefood matrix of mycoprotein would confer anabolic properties, resulting in a greater MyoPS response compared with the protein concentrate. In line with our previous work^(12,26)^, mycoprotein ingestion robustly increased resting and post-exercise MyoPS rates; however, contrary to our hypothesis, this stimulation was of a comparable magnitude following ingestion of the protein concentrate, implying no additive anabolic properties of the wholefood matrix.

We developed a novel protein concentrate with the aim of removing the protein from the wholefood matrix, thereby providing a direct comparison between protein delivered both *within* and *without* its wholefood matrix. This was a successful model as the removal of the non-protein constituents produced a concentrated protein source (73 %), resulting in divergent protein digestion and amino acid absorption kinetics (see Fig. 3 and 4) compared with the wholefood version. Despite this, however, we observed equivalent postprandial MyoPS rates across groups in both rested and exercised muscle tissue (see Fig. 6). In corroboration, we saw no differences in the muscle mTOR signalling response (Fig. 7) between conditions. The lack of differences observed in mTOR phosphorylation may be partially attributed to the muscle sampling timing, given that mTOR phosphorylation peaks 90–120 min following protein ingestion^(27,28)^ and the lack of assessment of downstream targets (p70SK1/4E-BP1). Nonetheless, collectively these data indicate that the wholefood matrix of mycoprotein did not augment the postprandial muscle anabolic response.

As expected, our novel protein concentrate increased the speed and magnitude at which leucine appeared in the plasma post ingestion compared with its wholefood counterpart (also observed in most amino acids measured and, resultantly, insulin; see Fig. 4 and 2). The rapid appearance of leucine and insulin in plasma in the PCM condition likely resulted in greater (and/or quicker) muscle leucine uptake, supported by the tendency for lower postprandial plasma leucine AUC. Therefore, despite leucine-matched conditions, the PCM bolus clearly provided a greater (and earlier) plasma and intramuscular leucine stimulus which the leucine trigger hypothesis would imply would confer a greater anabolic response^(29)^. Though the timing of the biopsies in the present study prohibited us from being able to detect a potential earlier (e.g. 2 rather than 4 h) stimulation of MyoPS and mTOR phosphorylaion in the PCM condition, our data contribute towards a growing number of studies that imply the plasma leucinaemic response is more, but not consistently so^(30,31)^, predictive of postprandial MPS rates when comparing consumption of isolated rather than wholefood protein sources^(12,13,26,29,32)^. However, taking the pivotal role of leucine at face value, it is interesting to speculate as to the role that the wholefood matrix may still have played in the stimulation of MyoPS in the present study. First, an indirect effect is plausible, whereby the wholefood/matrix sensitised the muscle to the amino acid/leucine stimulus, such that a lower peak was required to elicit the same MyoPS response compared with the isolated source^(10)^. Second, the wholefood and/or its matrix may have acted directly upon myocellular anabolic signalling pathways, providing additional (and independent) stimulation of MyoPS from the protein (leucine) alone. Either of these possibilities could plausibly still have been evident in the present work, allowing for an inferior postprandial leucine response to facilitate the same MyoPS.

Our data do not discount the possibility that a wholefood effect concerning postprandial muscle anabolism exists, considering these data are specific only to mycoprotein. However, it is of relevance to consider why a wholefood effect may not have been observable within the constraints of the present design. Although we produced a novel protein concentrate significantly purer in protein than the control condition (i.e. 73 *v*. 45 %), it was not a protein isolate (i.e. > 80 %) and, therefore, still contained non-protein constituents (Table 2). It is therefore plausible that the concentrate had retained enough of its wholefood nature to maximise any additional anabolic properties. Production of a purer mycoprotein isolate (i.e. > 80 %) would provide valuable insight into whether the concentrate produced in this study was pure enough to properly test the wholefood hypothesis. Alternatively, it is also possible that a maximal postprandial MyoPS response had been achieved simply by the amount of protein provided, rendering any additional effect of the wholefood delivery redundant. In support, postprandial rates of MPS following unilateral resistance exercise have been reported as maximal following the ingestion of 20 g of high-quality animal-derived proteins^(5,33)^ or, alternatively, roughly 2·5 g of leucine^(34)^. Participants in the present study consumed in excess of this protein amount in both conditions (31·5 g and 28·0 g in MYC and PCM, respectively) and also reached the suggested leucine threshold of 2·5 g. Given that previous evidence for the additional anabolic potential of delivering protein as part of a wholefood has been shown with 18 g of egg protein (roughly 1·6 g leucine)^(13)^ or 8 g of milk protein (roughly 0·8 g leucine)^(14)^, our data further this debate to imply that a potentiating anabolic effect of wholefood may only be present in response to suboptimal doses of protein. It therefore appears likely that any non-protein-related anabolic effects of a wholefood source would be alternative to (and not in addition to) the role of sufficient amino acids.

The present data, nor the preceding discussion, do not offer a satisfactory explanation for our previous observations concerning mycoprotein ingestion stimulating MPS rates to a greater extent than isolated milk protein despite optimal protein (> 20 g) and leucine (2·5 g) being provided in both conditions and greater leucinaemic responses in the milk condition^(12)^; neither proposed wholefood effects nor plasma leucine kinetics appear responsible. Rather, the present data point towards amino acid factors independent of leucine^(26)^ (e.g. total protein and/or specific amino acids) being of relevance. While human data are lacking, amino acids such as arginine, lysine and methionine are thought to be important from a myocellular signalling^(35–37)^ and substrate limitation perspective^(38–40)^. Intriguingly, there is also early *in vitro* evidence, suggesting that ergothioneine, a naturally occurring amino acid predominantly found in fungal-derived protein sources, can upregulate anabolic signalling pathways^(41)^. It will be of relevance for future work to establish optimal protein amounts with differing sources, as well as optimal amino acid compositions with consideration beyond leucine.

Finally, some broader context of the present data is worth discussion. Firstly, taking into account some of the wider metabolic impacts of ingesting protein within a wholefood matrix, we observed an attenuated insulin response following the ingestion of MYC compared with PCM (Fig. 2). While a rise in circulating insulin is necessary to support an increase in MPS^(42)^, regular peaks in insulin have been linked with the development of CVD^(43)^. Therefore, in the case of mycoprotein, it might be beneficial to consume as part of the wholefood matrix, given that this will robustly stimulate MPS while resulting in a lower peak in insulin. Secondly, considering the term ‘wholefood’ more specifically, it should be acknowledged that this encompasses a vast variety of food sources that vary in macro- and micro-nutrient content. Therefore, it is plausible, or even likely, that some food matrices may confer divergent anabolic effects. For instance, studies to date demonstrating an anabolic effect of wholefoods have used animal-derived food sources only^(13,14)^, rich in certain vitamins (e.g. vitamin D) and fat moieties (e.g. palmitate, cholesterol, *n*-3 fatty acids, phospholipids and phosphatidylcholine) that are suggested to modulate myocellular anabolic signalling pathways^(44–47)^. Conversely, it is suggested that the wholefood matrix of non-animal-derived sources (especially plant-based sources) is inhibitory to plasma amino acid appearance^(48–50)^, which may attenuate the postprandial MPS response. However, the present study is the first to investigate a wholefood effect of a non-animal-derived protein source. Our data clearly show that the wholefood matrix is not *inhibitory* to the MyoPS response following ingestion of mycoprotein, as could also reasonably be expected of a high-fibre protein source^(48)^. This highlights the complexity surrounding the ‘wholefood hypothesis’, suggesting that not all wholefood matrices possess the potential to potentiate (or attenuate) the MPS response. Instead, it is possible that specific nutrients within certain food sources (and delivery of these nutrients within a protein-dense food matrix) are key to a food source’s anabolic potency. Given the increasing drive to identify (or optimise) alternative (non-animal) dietary protein sources to support increasing global demands^(51)^, the future study of a wide range of protein-rich wholefood sources is clearly of paramount importance.

In conclusion, ingestion of leucine-matched boluses of mycoprotein or protein concentrated from mycoprotein results in equivalent stimulation of MyoPS rates in rested and exercised tissue, suggesting that one can consume protein within a wholefood or as an isolated source without compromising the anabolic response. These data demonstrate that the wholefood nature of mycoprotein does not confer additional anabolic capacity, at least when sufficient protein is provided.
